# A Novel Approach for Lidar-Based Robot Localization in a Scale-Drifted Map Constructed Using Monocular SLAM

**DOI:** 10.3390/s19102230

**Published:** 2019-05-14

**Authors:** Su Wang, Yukinori Kobayashi, Ankit A. Ravankar, Abhijeet Ravankar, Takanori Emaru

**Affiliations:** 1 Division of Human Mechanical Systems and Design, Faculty and Graduate School of Engineering, Hokkaido University, Sapporo 060-8628, Hokkaido, Japan; kobay@eng.hokudai.ac.jp (Y.K.); ankit@eng.hokudai.ac.jp (A.A.R.); emaru@eng.hokudai.ac.jp (T.E.); 2 School of Regional Innovation and Social Design Engineering, Faculty of Engineering, Kitami Institute of Technology, Kitami 090-8507, Hokkaido, Japan; aravankar@mail.kitami-it.ac.jp

**Keywords:** monocular SLAM, localization, scale drift, state estimation, heterogeneous robot system

## Abstract

Scale ambiguity and drift are inherent drawbacks of a pure-visual monocular simultaneous localization and mapping (SLAM) system. This problem could be a crucial challenge for other robots with range sensors to perform localization in a map previously built by a monocular camera. In this paper, a metrically inconsistent priori map is made by the monocular SLAM that is subsequently used to perform localization on another robot only using a laser range finder (LRF). To tackle the problem of the metric inconsistency, this paper proposes a 2D-LRF-based localization algorithm which allows the robot to locate itself and resolve the scale of the local map simultaneously. To align the data from 2D LRF to the map, 2D structures are extracted from the 3D point cloud map obtained by the visual SLAM process. Next, a modified Monte Carlo localization (MCL) approach is proposed to estimate the robot’s state which is composed of both the robot’s pose and map’s relative scale. Finally, the effectiveness of the proposed system is demonstrated in the experiments on a public benchmark dataset as well as in a real-world scenario. The experimental results indicate that the proposed method is able to globally localize the robot in real-time. Additionally, even in a badly drifted map, the successful localization can also be achieved.

## 1. Introduction

For a mobile robot operating in known or unknown areas, localization in a map of its work area is an essential requirement for several practical applications. In many cases, this prior map used for localization is generated through a simultaneous localization and mapping (SLAM) process, in which the robot builds the map of the unknown environment based on the information from sensors while estimating its pose at the same time. Various sensors, including optical cameras [1,2], ultrasonic wave sensors [3], laser range finders [4,5,6], or multiple sensors, can be used in SLAM systems depending on different applications and environments.

In the past years, the monocular SLAM, in which only a single camera is used, has been proven to be a desirable solution for building high quality maps with swift robots due to its simple hardware structure. However, monocular vision is not able to provide an absolute scale estimation of the landmarks. This leads to a problem that when performing monocular SLAM, the movement of the camera has to be fixed on an ambiguous scale factor. Furthermore, without a stable baseline between adjacent frames, this scale factor is also unstable and unreliable. Accordingly, the map built by monocular SLAM will suffer from metric inconsistency, which means that the scale of the map obtained by monocular SLAM is unobservable and gradually drifts over time.

With the help of a wealth of information provided by images, it is possible to perform localization in the map generated by monocular SLAM using another camera by leveraging the semantic features in the images. However, consider a typical multi-robot system in robot search and rescue applications, as described in [7]. Since the environment to be explored is always unknown and complicated, it is reasonable to send a swift “mapper” robot with a single camera first to give an overview of the target environment and build a map through monocular SLAM at the same time. Then, a “follower” unmanned ground vehicle (UGV), which has the load carrying capacity and often uses LRF for environment perception, can be deployed to the same area. By localizing themselves on the map provided by the “mapper” robot, the “follower” robots can carry out rescue activities more efficiently. It should be noted, in this scheme, that the “mapper” is only able to provide a map without the absolute scale. Hence, for the “follower” who wants to reuse this metric inconsistent map with only a range sensor, the unknown scale of the map could be a fatal drawback because the map without reliable range information is meaningless to the LRF and prevents the acquired sensor data from correctly associating with the map.

Recently, various methods have been proposed to solve the scale-drift problem in monocular SLAM. The most intuitive way to tackle the scale-drift problem is by fusing other types of sensors to make the metrical information in visual SLAM observable, e.g., the laser distance meter [8,9,10], IMU [11], or stereo system [12]. However, the most remarkable advantage of monocular SLAM is its portability. On the other hand, using additional sensors always means less compact, less power-saving and higher cost as compared to the one based on a pure monocular camera. Detecting the loop closure and drift scale recovery by using global bundle optimization [13,14] is also a well-studied approach for correcting scale drift. However, the problem is that the closed loop is not always permitted, and even if the loop can be successfully closed, the absolute scale is still unknown. In [15], the fixed height of the monocular camera is leveraged to impose the restrictions for scale correcting. Similarly, as proposed in [16,17], using the object recognition and size estimation of the items, the absolute scale can also be recovered during monocular SLAM.

All of the aforementioned works have provided constructive ideas and achieved ideal performances. It can be found that the goal of all of these techniques is to fix the scale drift in the map building process. In the present work, this problem is considered in another perspective: if a map has already been built by monocular SLAM, and the metrical inconsistency of a map is irreversible, is it possible to directly localize another robot in it with only an LRF while estimating the local scale at the same time? In the presence of the range information from LRF, new constraints can be introduced into the scale-drifted map, which makes the absolute scale of the map observable.

This issue is essentially a problem of localization in an “incomplete map”, which is defined as a map in which the constraints are either absent or incomplete. A few early works have been developed to address and solve a similar problem. Patric Jensfelt et al. [18] proposed an unsupervised method that learns the distance in a topological map built with errors. Kümmerle, Rainer et al. [19] created a SLAM framework utilizing aerial view images as prior information, in which the Monte Carlo localization (MCL) method has been employed to obtain the global constraints in the map extracted from aerial images. Mielle, Malcolm et al. [20] presented a graph-based localization method in an emergency map. They tackled the deformation of the emergency map by optimizing the shape of the matched graph. Sketch map drawn by hand is another typical metrically inconsistent map. Behzadian, Bahram et al. [21,22] converted a hand-drawn draft into pixel coordinate and utilized a local scale factor to approximate the deformation of the draft.

In this paper, we aim at designing an LRF-based method for localizing a robot in the map built from monocular SLAM. The application scenario of the proposed method is the multi-robot cooperation in a single-floor indoor space. The map obtained through the monocular SLAM process or visual odometry is a 3D point cloud, while the sensor used for localization is a 2D LRF. Therefore, the dimensionality of the point cloud is reduced by projecting it onto a 2D grid map to make the landmarks match the 2D scans. Then, to locate a robot in this map with scale ambiguity, the relative motion of the robot and the measurement of LRF are adjusted based on a flexible scale factor in order to make them adaptable to the size of the map. For achieving this goal, a variant of the MCL approach is presented to estimate not only the robot pose, but also the relative scale of the local map.

This paper is an extension of our previous work [23]. Our initial paper [23] only addressed the problem of localization in a relatively simple condition. In this article, a more detailed explanation of the proposed method is available. We also address the global localization problem and provide an in-depth evaluation of the extended MCL approach. Furthermore, experiments are conducted in a more challenging real environment to show the flexibility and robustness of the proposed method. The rest of this article is organized as follows: A general description of our proposed system and problem statement is given in Section 2. The detailed proposed methods are presented in Section 3 and Section 4. The experimental results are shown in Section 5.

## 2. System Overview

**Figure 1:**
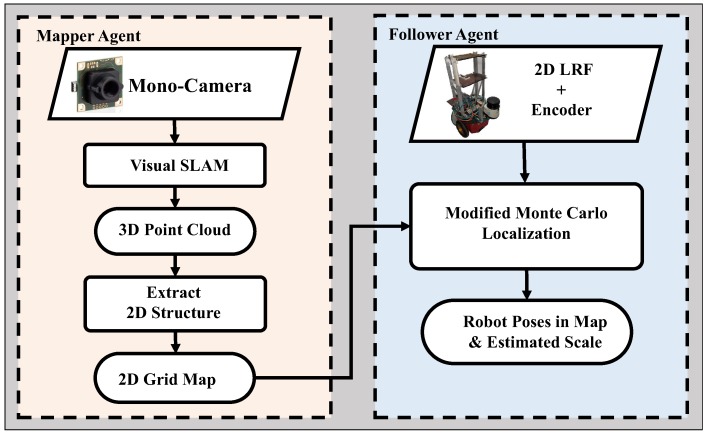
An overview of the proposed system, which is mainly composed of a “mapper” and a “follower” agent.

The outline of the proposed system is shown in Figure 1. The target environment is explored by a “mapper” agent, which can be a swift robot (UAV) or even a hand-held monocular camera. First, using the monocular SLAM, a 3D point cloud map is generated from the incoming image data and then pre-processed into a 2D representation. Next, a “follower” wheeled robot with LRF is deployed into the same area to perform localization in the constructed map. The LRF data, encoder data, and the map are inputs for the localization system. Whereas, the outputs are the local scale factor and the “follower” robot’s pose.

The idea behind our approach is based on the assumption that the scale drift in monocular SLAM is a gradual process, therefore, the local deformation of the built map is always insignificant and equal in every direction. In a local map of visual SLAM, a point set P is maintained to depict the landmarks L in the real-word. For the agent “mapper”, the absolute distance *d* between a landmark $l_{i}\in L$ and the camera center $O_{c}$ is unobservable. Instead, the monocular SLAM produces an arbitrary distance parameter $d_{e}$ between the corresponding point $p_{i}\in P$ and $O_{c}$ to give an estimate of *d* in the obtained map. For laser-based localization, the “follower” is sent to this certain local map, and it measures the same landmark $l_{i}$ with LRF from another perspective. The distance between the robot and landmark $l_{i}$ in the map is modeled as $d_{m}$. The actual distance, which is measured by the laser beam, is denoted as $z_{i}$. Under the previous assumption, the scale factor in the same local map should be invariable. Therefore, the local scale factor of the robot at time *t* is denoted as $s_{t}$:

(1) $$s_{t}=\frac{d_{e}}{d}=\frac{d_{m}}{z_{i}}+v_{s}$$

where $v_{s}$ is the noise of scale estimation. In Equation (1), *d* is unknown, while $d_{e}$ is given by the monocular SLAM and $z_{i}$ is the observation from the laser sensor. Hence, for a pose $x_{t}$ of the “follower” robot in the map frame, there exists a relative distance $d_{m}$ and its corresponding scale factor $s_{t}$, which describes the relationship between the drifted scale and the absolute one. Therefore, it is possible to search for the robot poses and local scale using the Monte Carlo method.

In this work, our main focus is on the development of a localization approach rather than addressing the problem of visual SLAM. The maps to be used in our system are extracted from the one built by the monocular SLAM method beforehand. In theory, our proposed localization algorithm is not confined to a specific SLAM method and works well on the point cloud maps generated by any monocular SLAM systems. For instance, in Section 5, our approach is experimentally evaluated on different types of 3D maps (semi-dense and sparse) built by various SLAM methods [1,2].

## 3. 2D Representation of the 3D Environment

In this section, our goal is to convert the 3D point cloud map into a 2D map in order to match the scans from 2D LRF; 2D structure extraction from 3D map is a technique commonly used for 2D–3D sensor fusion and data compression. Morton [24] presented a vertical structure representation method named the Strip Histogrim Grid, in which the scene is encoded as histograms and the objective identification is also enabled. A classifier named HLD is leveraged in [25]. It parameterizes the obstacles and classifies them into positive and negative ones in order to provide information for self-navigation. An improvement of the HLD (Height-length-density) classifer is given by Goeddel et al. [26], in which they assorted the obstacles based on the slope and developed a compact map structure. Other works proposed probability-based methods to handle the rasterization of 3D map. For instance, in [27,28], the Gaussian Mixture model is exploited to characterize the large-scale point clouds into a 2D set to achieve fast LiDAR localization.

For achieving the 2D representation, we employed the 2D occupancy grid map [29] where each location in the environment is assumed to be either occupied, free, or unknown. Here, the lines of sight from the camera center to the landmarks are considered as measurements. Every key frame from monocular SLAM is treated as an independent observation. Next, all the measurements from the camera are projected to a specified plane and the occupied probability of each grid is calculated using the approach employed in the laser-based mapping method [30,31,32].

**Figure 2:**
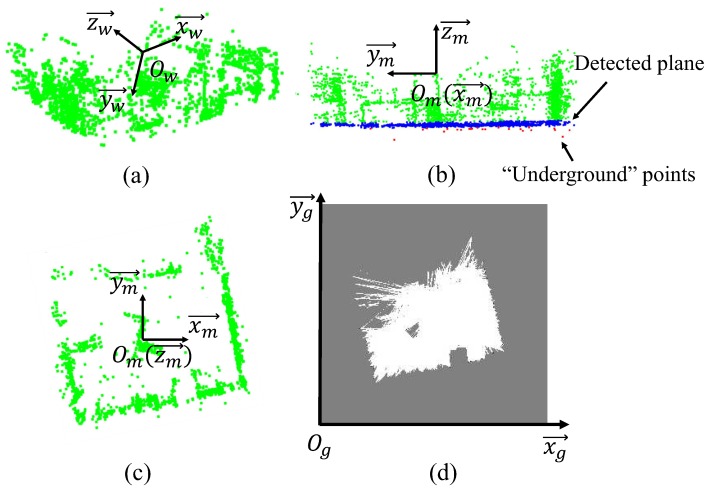
An example of 2D structure extraction on the EuRoC dataset [34]: (**a**) the raw point cloud built by monocular SLAM on the Euroc dataset, (**b**) floor plane detected by RANSAC and the new map frame $O_{m}$, (**c**) top view of the point cloud map, (**d**) extracted 2D grid map. The black, gray and white pixels represent the obstacle, unknown, and free grids, respectively.

Due to the relatively unknown camera absolute pose, it is necessary to decide the projection direction. For example, most indoor environment consists of flat floor surface that can be used to determine the coordinate system. To obtain the floor plane, RANSAC-based plane detection is applied to the point cloud map to extract the plane coefficients. However, the spurious planes that snatch parts of the points from the other items may lead to misidentification [33]. For instance, points of the planes segmented by the original RANSAC in the indoor environment frequently belong to parts of the edges of tables or chairs on the floor. The original RANSAC plane detection method is slightly modified by splitting all the 3D map points P with the hypothetical plane. We assume that the *y*-axis of the first camera frame, which is always defined as the *y*-axis of the world frame, should point to the ground. Since the points under the hypothetical plane can be considered as “underground” points that should not be observed by camera, the hypothesis plane with the maximum probabilistic $\hat{M}$ in the modified RANSAC is denoted as follows:

(2) $$\begin{array}{c} \hat{M}=argmax_{M}\left[\sum_{p_{i}\in P}\Gamma_{in}\left(p_{i},M\right)\right], \end{array}$$

where $\Gamma_{in}$ is the indicator of the plane model:

(3) $$\Gamma_{in}\left(p_{i},M\right)=\left\{\begin{array}{cc} 1, & |d_{i}|\leq d_{t}\\ -\mu , & |d_{i}|>d_{t}\;and\;F_{M}\left(p_{i}\right)>0\\ 0, & otherwise \end{array}\right.,$$

where $F_{M}\left(x\right)$ is the plane function of the hypothesis plane *M*. If the point-plane distance $d_{i}$ between the plane and point $p_{i}$ is within a threshold $d_{t}$, $\Gamma_{in}\left(p_{i},M\right)$ will return 1; if $p_{i}$ is an “underground” point below the hypothesis plane, $\Gamma_{in}$ will return a negetive value −μ. The parameter −μ is the weighting coefficient of the points under the hypothesis plane, which needs to be adjusted depending on different densities of the maps. In other words, the idea is to detect a plane with fewest points under it. Figure 2a provides an example of the point cloud from monocular SLAM on the EuRoC MAV dataset [34]. As shown in Figure 2b, the blue points are the inlier ones that belong to the floor plane extracted from the point cloud, and the “underground” points, which make a negative contribution to the weight of the detected plane, are marked in red.

The world frame of monocular SLAM is denoted as $\left\{O_{w},\vec{x_{w}},\vec{y_{w}},\vec{z_{w}}\right\}$, where $\vec{x_{w}},\vec{y_{w}},\vec{z_{w}}$ are base vectors of the world frame that is centred at $O_{w}$. Let $\vec{n}$ denote the normal vector of the plane obtained by RANSAC, which satisfies $\vec{n}\cdot \vec{y_{w}}>0$. Thus, the new map frame before projection can be defined as $\left\{O_{m},\vec{x_{m}},\vec{y_{m}},\vec{z_{m}}\right\}$, in which the unit vectors $\vec{z_{m}}$ is obtained by projecting $\vec{z_{w}}$ onto the detected plane $\hat{M}$; $\vec{z_{m}}=-\vec{n}$; $\vec{x_{m}}$ is the vector which is perpendicular to $\vec{y_{m}}$ and $\vec{z_{m}}$; $O_{m}$ coincides with the point $O_{w}$. Therefore, the transformation matrix from the world frame to the map frame is ${}^{m}T_{w}={\left[\vec{x_{m}},\vec{y_{m}},\vec{z_{m}}\right]}^{T}$.

If the resolution of the 2D grid map is ($X_{max}\times Y_{max}$), and *r* is the size of each grid (*m*/grid), the transformation from a world point $P=\{x_{p},y_{p},z_{p}\}$ to its corresponding 2D map grid $G=\{x_{g},y_{g}\}$ is given as:

(4) $$\left[\begin{array}{c} x_{g}\\ y_{g} \end{array}\right]=\left[\begin{array}{ccc} \frac{1}{r} & 0 & 0\\ 0 & \frac{1}{r} & 0 \end{array}\right]\left[\begin{array}{c} \vec{x_{m}}\\ \vec{y_{m}}\\ \vec{z_{m}} \end{array}\right]\left[\begin{array}{c} x_{p}\\ y_{p}\\ z_{p} \end{array}\right]+\left[\begin{array}{c} X_{max}/2\\ Y_{max}/2 \end{array}\right],$$

Consequently, the 3D map can be processed into a 2D grid map by calculating the occupancy probability of each grid. Furthermore, sometimes the camera FOV may go through spaces above the obstacles and therefore will not be able to observe them due to the limitation of the field of view. As a result, these unobserved landmarks may be regarded as paths which are free to pass. To get rid of this kind of misjudgement, a grid is considered as the constant obstacle if its occupied probability has already reached a threshold for a number of frames.

The top view of the point cloud built on Euroc dataset can be seen in Figure 2c. The extracted 2D grid map is shown in Figure 2d. Some of the landmarks and the shape of the target environment has been well described by the grid map. However, the left-top side of the grid map in Figure 2d is improperly marked as “unknown”. This is because the source map is generated by a sparse SLAM method [2], and the FOV of the camera in this dataset rarely focuses on this area.

## 4. 2D Laser-Based Localization in Scale Drifted Map

### 4.1. Extended Monte Carlo Localization

An extended MCL algorithm is employed to solve the LRF based localization problem. The extended MCL algorithm is built upon the conventional one proposed by Dellaert et al. [35,36] to make an extra estimation of the local scale factor $s_{t}$ at time *t*. We assume that $s_{t}$ only depends upon the position $x_{t}$ and the map *m*, the state of the robot can be updated by:

(5) $$\begin{array}{c} p(x_{t},s_{t}|z_{1:t},u_{1:t},m)\propto p\left(z_{t}|x_{t},s_{t},m\right)\cdot \int_{s_{t-1}}\int_{x_{t-1}}p\left(x_{t-1},s_{t-1}|z_{1:t-1},u_{1:t-1},m\right)\cdot \\ p\left(x_{t},s_{t}|x_{t-1},s_{t-1},u_{t}\right)dx_{t-1}ds_{t-1}, \end{array}$$

where, $x_{t}$ denotes the pose of the robot at time *t* in the map *m*, $z_{1:t}$ is the sensor observations. Given the control inputs $u_{1:t}$, the motion model $p(x_{t},s_{t}|x_{t-1},s_{t-1},u_{t})$ predicts the probability of updating the robot state into $\left\{x_{t},s_{t}\right\}$. The sensor model, or observation model, $p(z_{t}|x_{t},s_{t},m)$ denotes the likelihood of receiving sensor signal $z_{t}$ over the current state $x_{t},s_{t}$ in the map *m*.

In MCL, the probability of the robot states is randomly sampled and represented by a number of particles. Generally, the update of the belief is a three-step recursive process, also known as the particle filter:

1. Prediction step: A number of particles are sampled in line with the motion model $p(x_{t-1},s_{t-1},u_{t})$ to approximate the unknown robot state $\left\{x_{t},s_{t}\right\}$.
2. Correction step: All of the sampled particles are weighted according to the observation model $p(z_{t}|x_{t},s_{t},m)$. The details of the motion and observation models will be described in the following subsections.
3. Resample step: The algorithm samples new particles based on their weight. In this step, the particles are drawn with replacement, hence the ones with low probability will be eliminated, while the stability of convergence is also enhanced.

As discussed in Section 2, a prerequisite for achieving robust localization is the correct scale estimation. As a result, although the scale factor $s_{t}$ and robot pose $x_{t}$ are calculated by particle filter at the same time, the convergence of $s_{t}$ always precedes the robot pose $x_{t}$. In this case, the convergence process of the MCL method can be divided into two phases based on the behaviours of particles: (1) Scale factor initialization: the scale factor $s_{t}$ is first converged at a limited range. A parameter $\sigma_{c}$ is defined to judge whether the scale estimation is well converged. (2) Robot pose tracking: the pose of robot will be calculated only if the scale factor $s_{t}$ has already been converged, meanwhile, $s_{t}$ will also be continuously revised. Here, $\sigma_{c}$ is calculated by:

(6) $$\sigma_{c}=\sum_{i=1}^{n_{p}}{\left(\log s_{t}^{mean}-\log s_{t}^{i}\right)}^{2},$$

where, $n_{p}$ is the total number of employed particles at time *t*, $s_{t}^{i}$ is the scale factor of the *i*-th particle at time *t*, $s_{t}^{mean}$ is the average scale factor of all the particles at time *t*. The scale factor will be considered as converged only if the $\sigma_{c}$ is smaller than 0.8 for 5 updates continuously. This threshold value is determined empirically.

### 4.2. Motion Model

**Figure 3:**
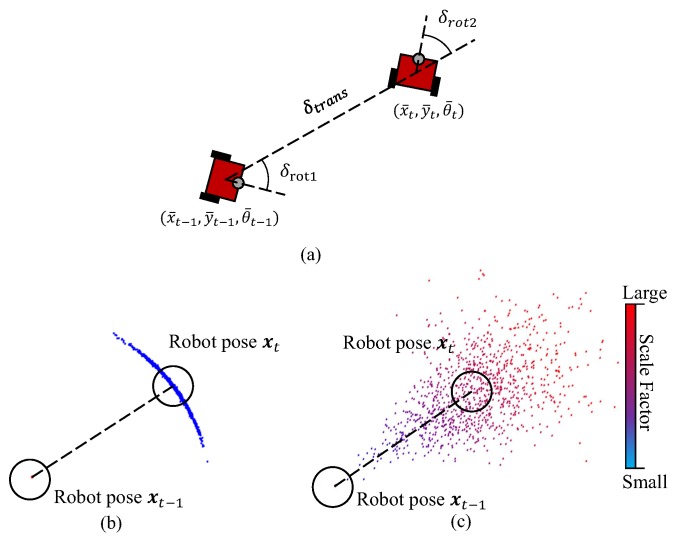
(**a**) motion model of the robot, (**b**) sampling 1000 particles from the classical motion model, (**c**) sampling 1000 particles from the motion model with scale uncertainty. The colors of the points are used to represent the specified scale factors. The darker color represents smaller scale factor $s_{t}$.

The task of the robot motion model is to compute the posterior distribution $p(x_{t},s_{t}|x_{t-1},s_{t-1},u_{t})$. In our work, the odometry model based on the encoder will be used to predict the motion. Figure 3a illustrates the robot odometry model. When the robot advances from ($x_{t-1},y_{t-1},\theta_{t-1}$) to ($x_{t},y_{t},\theta_{t}$), the relative motion can be indicated by a parameter vector $\delta_{m}=\left\{\delta_{trans},\delta_{rot1},\delta_{rot2}\right\}$, in which the element $\delta_{trans}$ is the translation, $\delta_{rot1}$ and $\delta_{rot2}$ are the first and second rotation of robot head. Given the reading of encoder, $\delta_{m}$ can be computed as:

(7) $$\left\{\begin{array}{c} \delta_{trans}=\sqrt[{}]{{\left({\bar{x}}_{t}-{\bar{x}}_{t-1}\right)}^{2}+{\left({\bar{y}}_{t}-{\bar{y}}_{t-1}\right)}^{2}}\\ \delta_{rot1}=atan2\left({\bar{y}}_{t}-{\bar{y}}_{t-1},{\bar{x}}_{t}-{\bar{x}}_{t-1}\right)-{\bar{\theta }}_{t-1}\\ \delta_{rot2}={\bar{\theta }}_{t}-{\bar{\theta }}_{t-1}-\delta_{rot1} \end{array}\right.,$$

where, $\left\{{\bar{x}}_{t},{\bar{y}}_{t},{\bar{\theta }}_{t}\right\}$ is updated by the raw measurement of the encoder. Through modelling the motion error under Gaussian distribution, the rotation and translation can be sampled by:

(8) $$\left\{\begin{array}{c} {\hat{\delta }}_{trans}=\delta_{trans}-G\left(\alpha_{3}|\delta_{trans}|+\alpha_{4}|\delta_{rot1}+\delta_{rot2}|\right)\\ {\hat{\delta }}_{rot1}=\delta_{rot1}-G\left(\alpha_{1}|\delta_{rot1}|+\alpha_{2}|\delta_{trans}|\right)\\ {\hat{\delta }}_{rot2}=\delta_{rot2}-G\left(\alpha_{1}|\delta_{rot2}|+\alpha_{2}|\delta_{trans}|\right) \end{array}\right.,$$

where, the function G(b) means Gaussian noise with variance *b* and zero mean. $\alpha_{x}$ denotes the motion error parameter of the robot, which specifies the error of robot state accrued with the pose changing, similar to the defination in [29].

Additionally, the scale factor $s_{t}$ should also be updated with noise. It has been observed that one of the main reasons of the scale drift is the large rotations of camera. As a result, serious scale drifts frequently occur at the corners in the map. In this case, the rotation is taken into account for the prediction of $s_{t}$ in the motion model. The $p(s_{t}|s_{t-1})$ is defined as a Gaussian distribution:

(9) $$s_{t}∼N\left(s_{t-1},{\left(\sigma_{s}+\alpha_{5}\left(\delta_{rot2}+\delta_{rot1}\right)/\pi \right)}^{2}\right),$$

where $\left(\delta_{rot2}+\delta_{rot1}\right)$ is the angle of robot’s rotation between *t* and t−1, and $\sigma_{s}$ is the standard deviation of variable $s_{t}$ of the particles in the cluster with the highest weight.

Then the pose of robot can be updated by:

(10) $$\left\{\begin{array}{c} x_{t+1}=x_{t}+s_{t}\cdot {\hat{\delta }}_{trans}\cdot \cos\left(\theta +{\hat{\delta }}_{rot1}\right)\\ y_{t+1}=y_{t}+s_{t}\cdot {\hat{\delta }}_{trans}\cdot \sin\left(\theta +{\hat{\delta }}_{rot1}\right)\\ \theta_{t+1}=\theta_{t}+{\hat{\delta }}_{rot1}+{\hat{\delta }}_{rot2} \end{array}\right.,$$

Figure 3b,c depicts the distributions of 1000 samples from the classical odometry model and the one with uncertain scale factor. In these two figures, the sampling has been done under the same parameters of motion model, however, it can be seen that our model is with higher uncertainty owing to the metrical inconsistencies. Therefore, the proposed localization method usually takes longer time to be converged in contrast to the original MCL method.

### 4.3. Sensor Model

The beam model, or the laser ray model [29], is utilized to calculate the importance of each particle. Intuitively, the working principle of beam model in a MCL algorithm is to fix the laser scanner onto the poses of all the particles in the map *m*. These hypothetical beams are cast along their current directions until hitting obstacles or reaching their maximum ranges. The weight of each particle will be determined based on the degree of agreement between these beams and the map *m*. Let the *i*-th measurements on map *m* be $z_{t}^{i}$. The likelihood of the full scan is composed of all the $n_{z}$ beams:

(11) $$p\left(z_{t}|x_{t},s_{t},m\right)=\prod_{i=1}^{n_{z}}p\left(z_{t}^{i}|x_{t},s_{t},m\right),$$

where, the model of a single beam incorporates four error types sourcing from four different conditions (correction detection, unexpected obstacles, missed measurements, and random measurements):

(12) $$p\left(z_{t}^{i}|x_{t},s_{t},m\right)={\left(\begin{array}{c} \phi_{hit}\\ \phi_{short}\\ \phi_{max}\\ \phi_{rand} \end{array}\right)}^{T}\cdot \left(\begin{array}{c} p_{hit}\left(s_{t}\cdot z_{t}^{i}|x_{t},m\right)\\ p_{short}\left(s_{t}\cdot z_{t}^{i}|x_{t},m\right)\\ p_{max}\left(s_{t}\cdot z_{t}^{i}|x_{t},m\right)\\ p_{rand}\left(s_{t}\cdot z_{t}^{i}|x_{t},m\right) \end{array}\right),$$

where, $\phi_{hit},\phi_{short},\phi_{max},\phi_{rand}$ are the weights defined for averaging the four types of distributions. The method to calculate $p_{hit},p_{short},p_{max}$, and $p_{rand}$ is identical to the original beam model described in [29].

Since the map built by monocular SLAM cannot perfectly depict the target scene, some free space gets marked as obstacles by mistake. Therefore, the particles are allowed to travel through the unreachable parts in the grid map. However, this will cause a new problem that some particles may pass the “walls” in the map and increase the risk of localization failure. Therefore, the weights of the particles in the unknown grids are penalized by multiplying a coefficient $\lambda_{u}=0.9$. Similarly, the penalty coefficient for the particles in the occupied grids is $\lambda_{o}=0.4$.

### 4.4. Initialization of Particle Filter

At the start of the MCL approach, the particles need to be set based on an initial guess of the robot’s state. Since the size of the original map is unknown, the initial scale factors of all the particles are randomly set in a range obeying uniform distribution. For example, if the particles are initialized with scale factors in the range [0.01 m/grid, 3 m/grid], a grid map with the size 640 × 640 will be able to represent the environment with a dynamic size from 6.4 m × 6.4 m to 1920 m × 1920 m, which covers all the possible sizes of the map for a typical indoor environment.

Furthermore, if the robot has no knowledge about its starting position, it is necessary to estimate its pose in the entire map, which is known as the global localization problem. In this work, one of the key advantages of using MCL algorithm is the ability to handle the global localization by distributing particles over all the possible grids in the map. For the evaluations in Section 5, the method is tested with a manually set initial guess around the starting point, and also under a global distribution covering all the possible hypotheses states for global localization.

During the initialization process, a large number of particles are required for searching among all the possible scale factors. However, after a few steps, most of the particles with incorrect scales will be eliminated in the resampling step and the variance of *s* will also be decreased accordingly. Then, the performance of particle filter in our method become similar to the original MCL, thus tracking of robot pose can be done with much fewer particles. To determine the number of particles, the KLD (Kullback-Leibler divergence) sampling approach [37] is utilized, which gives an approximate estimation of the KL divergence between the true distribution and the estimated one. With the help of KLD sampling, the computational cost of our approach is reduced to ensure the algorithm to be able to achieve the real-time level.

### 4.5. The Problem of Particle Collapse

In the resample process of the particle filter, if only a small number of particles carry the vast majority of weights whereas the rest of the particles have the minimum weights, the diversity of particles will be reduced. This may lead to a situation where the algorithm may fall into a single peak, which is a common problem in the particle filter, known as the particle collapse. Normally, this can be solved by setting trigger conditions for the resampling and applying low variance sampling.

**Figure 4:**
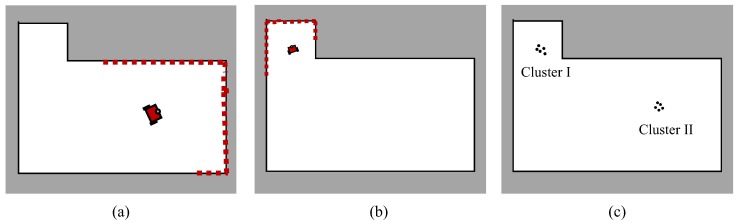
An example scenario where particle collapse may happen: (**a**,**b**) depict two different robot states which are about equally possible according to the sensor model. (**c**) shows two clusters of particles generated at the same moment, around the hypothesis states.

Nevertheless, in our case, the uncertain scale could also be an underlying cause of the particle collapse. For instance, Figure 4a,b shows two possible robot states carrying different scale factors but observing similar laser measurements with different poses. With similar probabilities, two clusters of particles will be generated around these two positions as shown in Figure 4c. Assume that the Cluster I in Figure 4c represents the correct robot pose. If Cluster I is eliminated after a few updates, all the resampled particles will be incorrectly gathered at the Cluster II, that is how the particle collapse happens.

In our practical tests, the proposed method is prone to fall into local optimal with very small scale factors because of the ambiguity of the map scale, especially in the global localization. To avoid this collapse problem, the candidate particles in a few sub-optimal clusters should be prevented from being discarded to make the system more robust against the multi-optimal situations, similar to the idea in [38]. A kd-tree is maintained for storing all the particles in each update. Then, we cluster the particles according to the distance in {x,y,θ} into several clusters and then sort them on their total weights. A number of clusters with the highest weight are selected for tracking multi-model hypotheses in the initialization step.

In the clustered MCL [38], several clusters of particles are updated independently for global trace, which is not required for our case. Instead, our strategy is to put a part of the particles in each selected cluster into a “safe box” and keep them from being abandoned during the resample operation. Moreover, particles in all the clusters are involved in the same MCL process, which means that these clusters are dynamically changed in every update. Whereas, this operation will introduce bias to the system owing to artificially increasing the probability of the states carried by the particles in the “safe box”. Hence, this method is only applied until the scale is considered as converged.

## 5. Experimental Evaluation

In this section, the performance of our proposed localization method is evaluated on two datasets. First, a group of tests is given on a public benchmark dataset, the MIT Stata dataset [39], in which data from both 2D LRF and camera is included. Then, the method is evaluated on our own devices in a real-world environment.

### 5.1. Results on Benchmark Dataset

#### 5.1.1. Experiment Setup

**Figure 5:**
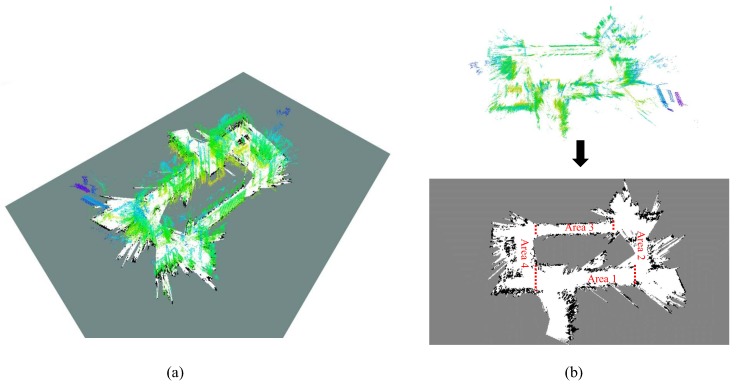
Map of the target environment, built based on the sequence *ms-38* of the MIT Stata dataset [39]. (**a**) The point cloud map that is transformed to the map coordinate and its 2D projection. (**b**) The extracted 2D grid map. The same experimental setup has also been presented in our previous conference paper [23].

Three sequences, the *2011-01-19-07-49-38*, *2011-01-21-09-01-36*, and *2011-04-06-38-27* (referred as sequence *ms-38*, *ms-36*, *ms-27* henceforth, respectively) in the MIT Stata dataset [39] are used. Since these sequences are acquired in the same building, it is possible to simulate all the necessary tasks, including the monocular SLAM process and localization tests, with these sequences. First, the LSD-SLAM [1], a popular semi-dense monocular SLAM framework, is applied on the image data in *ms-38* to obtain a 3D map and its 2D-grid projection. Then, using the laser data in *ms-38*, *ms-36*, and *ms-27*, the proposed scale-aware localization approach is performed in the obtained map. The point cloud map built by monocular SLAM and its 2D projection is shown in Figure 5. The map is divided into several areas and label them with different area numbers for convenient description.

In all these experiments, including the ones on the real dataset presented in the next subsection, the same set of parameters for the MCL method are used. We set $\left\{\alpha_{1},\alpha_{2},\alpha_{3},\alpha_{4},\alpha_{5}\right\}=\left\{0.1,0.5,0.8,0.5,0.01\right\}$ for the motion model; and $\phi_{hit}=0.5,\phi_{rand}=0.1,\phi_{short}=\phi_{max}=0.05$ for the observation model.

#### 5.1.2. Evaluation of Pose Tracking

First, in order to test only the tracking and scale estimating performance, the global localization is not taken into account. Therefore, the rough location of the robot’s starting point is considered as a known condition and the initial samples of the robot’s pose will be generated in a wide area around its starting position, under a normal distribution. First, the proposed method is tested with the particle number 1000–3000. This interval indicates that the lower limit of the particle number for KLD sampling is set to 1000, and the maximum number of the employed particles is 3000, which is also the particle number in the first iteration of the particle filter.

**Figure 6:**
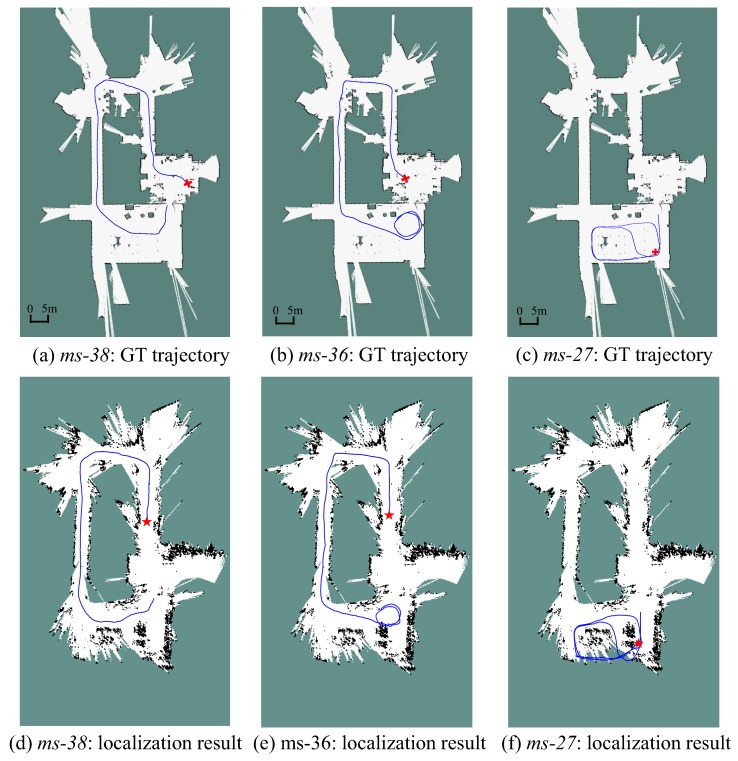
Real-time self-localization trajectories on the MIT Stata benchmark dataset. The first row plots the trajectory with ground truth. The second row depicts the trajectories estimated by the proposed localization method. The red cross marks in (**a**–**c**) are the initial locations of the robot. The red star marks in (**d**–**f**) are the locations where MCL method is converged (also the position where the trajectories start to be recorded).

The MCL algorithm is a highly non-deterministic process. Therefore, we performed 5 runs for each sequence. The trajectories estimated by the proposed localization approach on the three sequences are plotted in Figure 6. Here, a comparison between the 2D grid map from monocular SLAM and the one built by the LRF [40] with correct scale is provided. The ground truth path is shown in Figure 6a–c for comparison. The trajectories from Figure 6d through Figure 6e are recorded after the particle filter are converged in scale. Both the *ms-38* and *ms-36* start in the “Area 1” and end in the “Area 4” (the area division is referred to Figure 5), while the active range of *ms-27* is within the “Area 4”. It can be noticed that although the grid map is deformed in contrast to the LRF built map, the shapes of the trajectories still tally with the ground truth. A video of the localization process can be found in the supplementary section.

**Figure 7:**
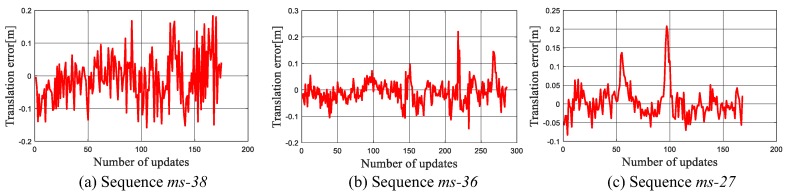
The translational errors $e_{trans,t}$ in the localization tests on MIT Stata dataset.

In the MIT Stata dataset, ground truth of mobile robot’s poses in all the sequences is provided. It is difficult to directly evaluate our experimental result based on it because the ground truth is recorded in the real world coordinate, while our experiments are carried out in the deformed map. Therefore, we turn to validate the error of the estimated translation between $\left\{x_{t},s_{t}\right\}$ and $\left\{x_{t-1},s_{t-1}\right\}$, which is defined as:

(13) $$e_{trans,t}=\frac{x_{t}-x_{t-1}}{s_{t}}-x_{t}^{g}+x_{t-1}^{g},$$

where, $x_{t}^{g}$ is the robot’s ground truth pose at time *t*. Both the localization and scale estimation accuracy can be accessed by the translational error because once the localization fails or the scale factor is estimated incorrectly, abrupt changes will appear on $e_{trans,t}$. The translational error $e_{trans,t}$ on three sequences are plotted in Figure 7. It can be seen that the error fluctuates within a narrow range. This shows that our localization method provides a reliable state estimation for the robot.

**Figure 8:**
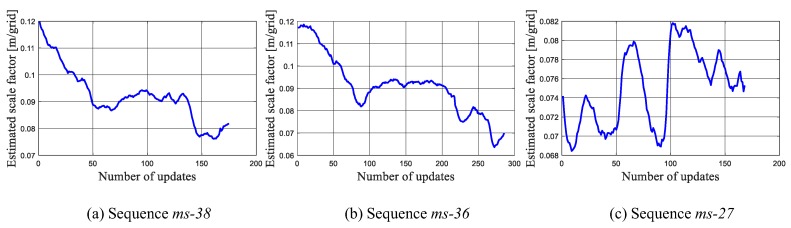
The estimated scale factor in the tests on the benchmark dataset.

The scale estimation results of a group of runs on three sequences is shown in Figure 8. Since *ms-38* and *ms-36* are acquired following the same path, Figure 8a,b show a similar trend, starting from 0.12 m/grid and then drifting to a small value gradually. The *ms-38* and *ms-36* ends in the “Area 4”, which illustrates that the scale factor in “Area 4” is around 0.08 m/grid. This is also in consistent with the result shown in Figure 8c, in which the scale factor in “Area 4” is estimated to be within the range from 0.068 m/grid through 0.082 m/grid.

**Figure 9:**
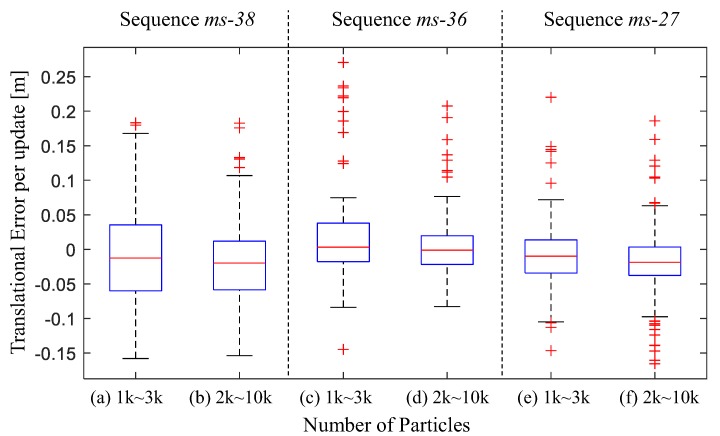
The translational errors in the tests with different amount of particles.

The number of particles is one of the influencing factors of the MCL method. Hence, it is worth discussing the state estimation performance with different particle numbers. Another group of tests is performed with a larger particle number: 2000–10,000. A comparison between the translational errors in the tests with different particle numbers are depicted in Figure 9. Some data of the localization results is summarized in Table 1. In this table, the translational error rate is calculated by $[\sum_{t=0}^{t_{end}}∥\left(e_{trans,t}\right)/\left(x_{t}^{g}-x_{t-1}^{g}\right)∥]/n_{end}$, where $t_{end}$ stands for the final update, and $n_{end}$ denotes the the number of updates.

Figure 9 verifies that the larger amount of particles contribute to the higher accuracy. This is also supported by the data in Table 1. Furthermore, Figure 9 also demonstrates that the boxplots of the tests with 1 k–3 k particles is more likely to be skewed to the right, which indicates that the pose estimation with fewer particles drifts more frequently.

Table 1 illustrates that, the proposed method is able to provide effective state estimations on *ms-36* and *ms-38* for all these runs. For the *ms-27*, because of the incompleteness of the map of “Area 4”, the robot has to pass through the “unknown” grids as shown in Figure 6f. This may lead to the localization failures and greater error. At the beginning of sequence *ms-36*, the robot stays statics for about 10 s, therefore the convergence speed is slower as compared to other sequences. Note that, the larger number of particles lead to a greater particle diversity as well, therefore, sometimes the particle filter with 1–3 k particles has faster convergence ability.

#### 5.1.3. Global Localization Tests

If the robot starts off with no idea of its position relative to the map, the MCL method needs to be initialized as a global localization problem. In our case, the global localization can be achieved by initially distributing the state hypotheses over all the possible poses and scale factors. Since more particles are needed for the global localization, 2000–10,000 particles are employed in the global localization tests. Here, it should be noticed that in consideration the incompleteness of the map, the particles are initially generated not only on the free grids (white grids), but also in the unknown parts (gray grids).

**Figure 10:**
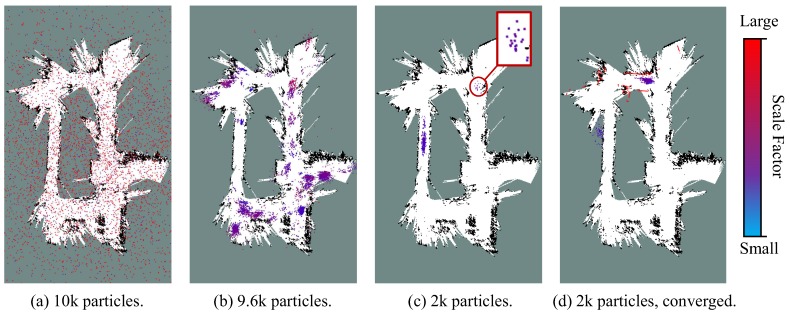
Particle distribution during the initial stage of the global localization process on the sequence *ms-38*. The scale factor of the particles are indicated by their colors. (**a**) The initial distribution of the particle filter. (**b**) The particle filter yields a highly diverse range of particle clusters after a few seconds. (**c**) A small number of clusters survive after several updates. The correct robot state is represented by the cluster labelled with the red circle. (**d**) The particle cloud is converged, suggesting that the scale estimation has been converged.

Figure 10 quantitatively depicts a converging process of the particle filter in the test of global localization on *ms-36*. The color of a particle represents its scale factor. In the first iteration (Figure 10a), particles with varying poses and scale factors are randomly sampled. Within seconds (Figure 10b), the scales of particles is rapidly converged into a small range. However, there still exist multiple candidate clusters centred at different poses. Soon afterwards, there are only a few clusters left (Figure 10d). The red circle indicates the cluster which should be the optimal solution, whereas it carries very low weight. Finally, at the end of initialization phase (Figure 10d), the particles are successfully localized in the same cluster with the similar scales, and the state estimation of the robot will be considered as converged.

The global localization performance on the sequence *ms-38* and *ms-27* is evaluated as well. The state estimation in all these tests can be initialized successfully, however, with significant longer convergence time. After the global localization is converged, the performance of the MCL method will be identical to a pose tracking process.

### 5.2. Results in Manually Collected Experiments

**Figure 11:**
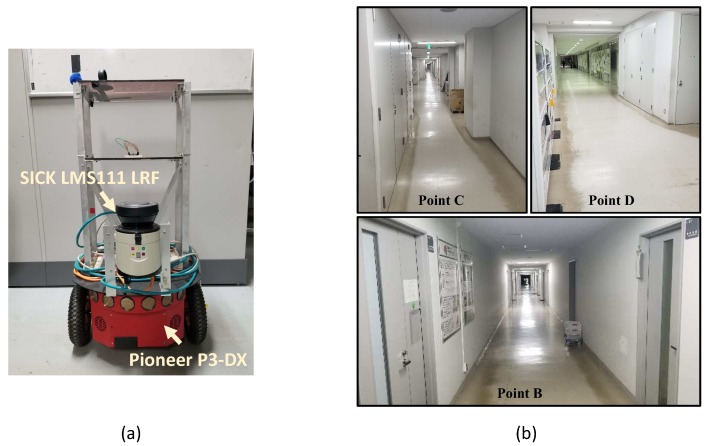
(**a**) Pioneer P3-DX used to perform localization. (**b**) Experimental environment, the engineering building of Hokkaido University.

To test the feasibility of our proposed method, experiments are performed in real environments with a ground robot. In our practical experiments, a hand-held web camera paired with a wide-angle lens is used to acquire the image data and perform the mapping. A Pioneer-P3DX wheel robot as shown in Figure 11 is used as the experimental platform to perform localization on the obtained map. The robot is equipped with a 2D LRF at about 0.3 meters above the ground. The average speed of the robot is about 1.2 m/s. Several key parameters of the camera and the LRF are displayed in Table 2 and Table 3 respectively. The scan from LRF is uniformly downsampled to 40 beams according to their emitting angle.

**Figure 12:**
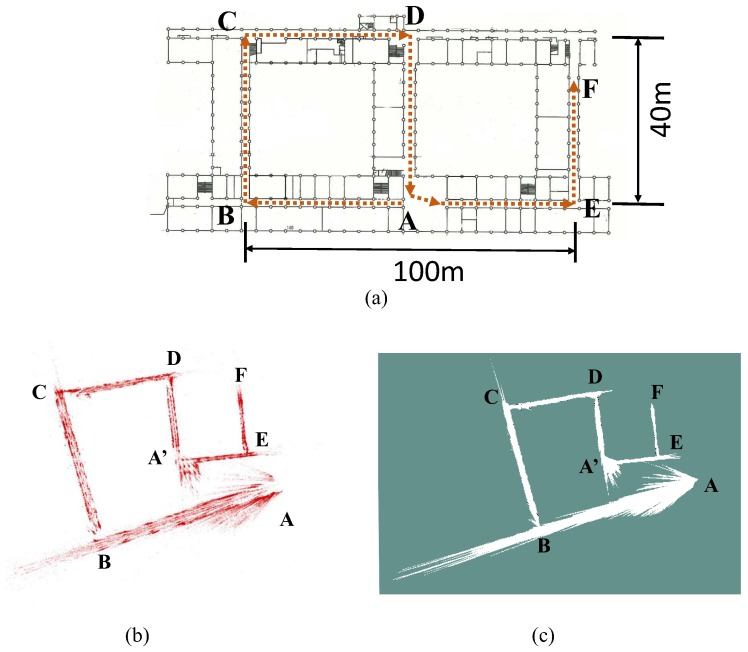
(**a**) The plan of the experimental environment. Red dot arrows represent the direction of camera trajectory, (**b**) scale-drifted point cloud map obtained by monocular SLAM, (**c**) 2D grid map. In (**b**,**c**), point A and A’ should be at the same position if the map is metrically correct.

A floor plan of the target environment with the correct size is shown in Figure 12a. To collect the image data, we hold the camera and walk from point A to F, the travel path on the floor plan is a S-shape one depicted as the red dashed lines in Figure 12a. The monocular version of ORB-SLAM2 [2] without loop closure is employed for visual mapping. The obtained raw map is shown as the point cloud in Figure 12b. Compared with the real path in Figure 12a, it can be noticed that significant scale drift occurs during the SLAM process, and the shape of the map is distorted. This also reinforces the fact that the scale of monocular SLAM drifts more greatly when a large rotation occurs [14]. In the obtained map, point A and A’ should be the same point if the map is metrically consistent or the loop-closure is carried out. As the result, the point A is not directly linked to point E. Finally, the raw map is processed into a 2D grid one for performing localization as shown in Figure 12c.

Next, the P3-DX is controlled to move following the identical path of the camera and collect a sequence of LRF data named *hu-1*, from point A to F. Then, the same robot is driven along the opposite direction, from point E to A, to collect another sequence named *hu-2*. The localization tests are performed based on both the two sequences.

**Figure 13:**
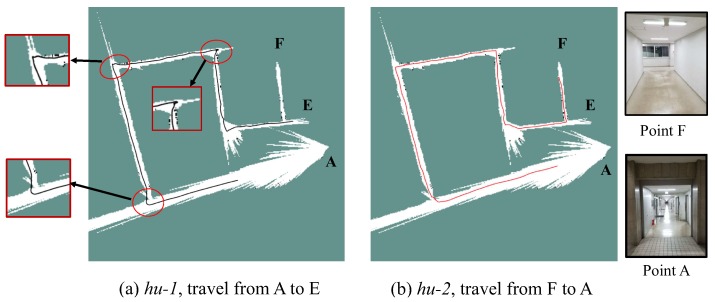
Estimated trajectories on the manually collected dataset. The red circles highlight the distorted parts caused by the accumulated error. Snapshots at point A and F are shown at the right side.

In this experiment, the particle filter is initialized around the positions where the robot starts off. The number of particles is set as 2000–6000. The estimated trajectories of both the two sequences are plotted in Figure 13. Because of the low quality of the map around point A, it takes 12 s for the *hu-1* to be converged into a single solution, while the *hu-2* is more rapidly converged in 4 s. Five runs are performed on the two sequences respectively. The proposed method is able to provide correct localization result on all these runs. In rare occasions, the particles spread out along different directions as multi-model distributions.

It may be found that the trace is distorted at the position as labelled by the red circles in Figure 13a. This is because of the lack of geometry features in the corridors that prevents the laser measurement model from correcting the error of the estimated displacement along the corridor. This displacement error will be accumulated until the robot comes across a corner and thereby lead to these distortions of traces. This issue can hardly be fixed depending on only the LRF, even though, the proposed MCL is still able to re-converge and output the correct trajectories.

**Figure 14:**
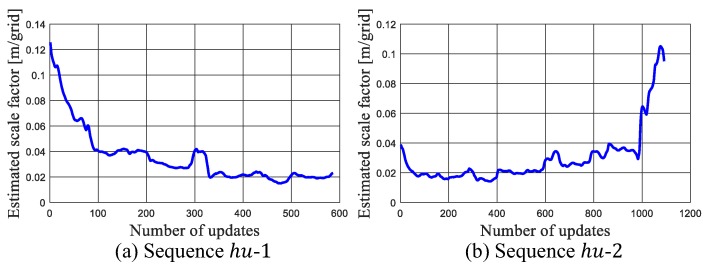
Estimated scale factor in the tests on manually collected dataset.

Then, the effect of scale estimation during localization is investigated. Figure 14 shows the estimated scale factors in these tests. It can be seen that the trends of the two sequences are symmetric to each other since they travel along opposite directions. The scale factor at point A is around 0.9, and drifted to 0.2 after reaching point F.

**Figure 15:**
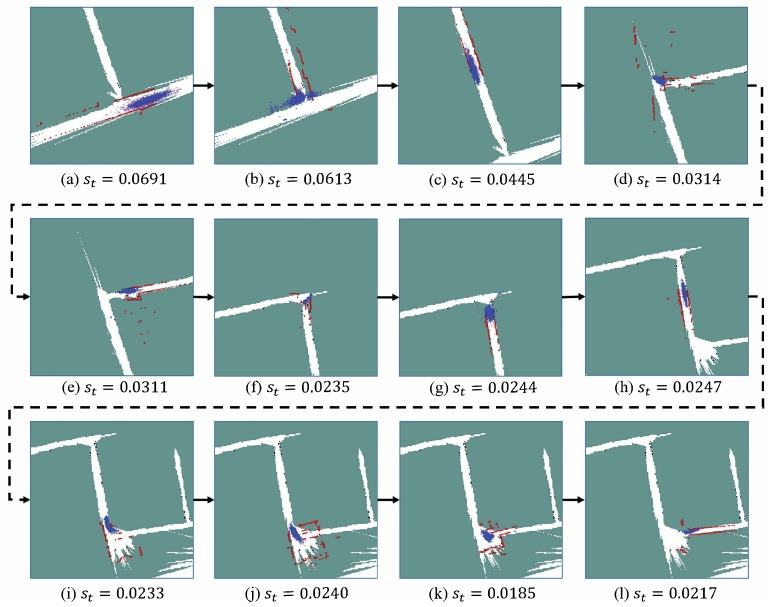
The scale correction for making rescale measurements match the drifted map. Red dots are the laser beams that are rescaled based on the estimated $s_{t}$. Blue points are the particles employed by the MCL method.

Figure 15 describes the processes of how the scale factor is adjusted to align the drifted map. The red points shown in Figure 15 are the laser points that are rescaled based on the estimated scale factors $s_{t}$. It can be seen that once the robot comes across a corner, there is a large disagreement between the laser beam and the map. After being corrected by the measurement model, the particle filter updates the $s_{t}$ in order to adapt the LRF measurements to the map. In the experiments, these trajectories are visually inspected in order to ensure that no localization failure happens. The experiments illustrate that our proposed method is able to withstand the large scale drift in the map of the low-texture environment. Moreover, the video of the practical experiments can also be found in the Supplementary Section.

### 5.3. Runtime Performance

**Figure 16:**
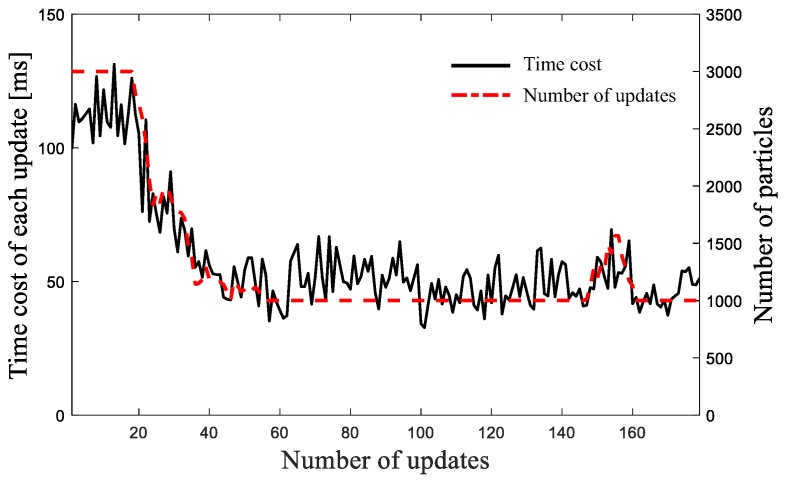
Runtime performance of the extended MCL algorithm’s each iteration.

All the evaluations were carried out on a PC with Intel Core i7-3740m CPU and 8 GB memory. Figure 16 shows the time cost of the proposed 2D localization algorithm at each update. As discussed in [23], the computational cost of the particle filter is proportional to the number of particles. The main cost of an update is the calculation of the observation likelihood based on the beam model and the resample process. In the beginning, because of the high uncertainty of the robot state, a large number of particles are required, hence it takes about 130 ms to manipulate 3 k particles. Then a steep reduction of the time cost can be observed owing to the effect of KLD-sampling. Finally, the particle number reaches the low limitation, and the time is optimized at about 40 ms. Moreover, the time cost of each update is around 350 ms with 10 k particles, which is the maximum computational cost of all the evaluations in Section 5. It is of note that the particle filter is not updated under a fixed frequency. Instead, the update is triggered only if the robot makes a certain transformation. Under the moving velocity of our robot platform (0.6 m/s), the average time interval between two updates is about 390 ms, thus the real-time performance can be achieved at most of the time.

## 6. Conclusions

In this study, we propose a novel localization approach which allows the robot to reuse the scale-drifted map from monocular SLAM, with only a laser range finder. To the best of our knowledge, the framework proposed in this article is the first trial to address the problem with this setup. By extracting 2D structures from the point cloud, the 3D point cloud from monocular SLAM is converted to a 2D grid map for localization. Our proposed localization approach is built upon a variant of Monte Carlo localization method, where both the robot’s pose and map scale are handled. The proposed method is tested on a public dataset and real environments. The result shows that the localization method can provide a relatively accurate estimation of the robot pose. Moreover, in feature-less environments, where the monocular SLAM produces significant scale drift, our method is still able to give a quick and consistent localization result with correct scale estimation.

In the future, the current work can be fulfilled in multiple directions. Some limitations of the proposed method have already been discussed, we believe that utilizing a more robust measurement model, such as likelihood field model [41] or normal distribution representation [42], may be a possible solution for enhancing the localization accuracy. Also, the pose estimation can be extended to 6 DoF that the usage of 3D LIDAR sensors will be involved in order to improve the robustness against the point cloud map. Futrthermore, the auto-navigation can also be achieved via combining the existing path planning method [43].

## Figures and Tables

**Table 1:** Performance on the MIT Stata dataset.

	Particle Number: [1 k, 3 k]			Particle Number: [2 k, 10 k]		
	*ms-38*	*ms-36*	*ms-27*	*ms-38*	*ms-36*	*ms-27*
Success rate	100 %	100%	40%	100%	100%	100%
Translational Error [%]	25.9	21.1	15.1	19.2	14.3	15.0
Convergence time [s]	25	23	6	28	22	19

**Table 2:** The camera used in experiments.

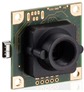	**Model**	**Resolution**	**Frame Rate**	**Shutter**	**Angle of View**	**Focus Length**
	UI-1221-LE	752×582	30 Hz	Global Shutter	130°	2.4 mm

**Table 3:** The LRF used in experiments.

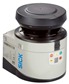	**Model**	**Aperture Angle**	**Scan Frequency**	**Resolution**	**Min. Range**	**Max. Range**
	LMS-111	270°	25 Hz	0.25°	0.5 m	20 m

## Supplementary Materials

The videos with experimental results are available online at https://www.mdpi.com/1424-8220/19/10/2230/s1.
